# Inflammation-Based Hematologic Indices as Prognostic Markers in Pulmonary Arterial and Chronic Thromboembolic Pulmonary Hypertension: A Hypothesis-Generating Registry Study

**DOI:** 10.3390/ijms262210940

**Published:** 2025-11-12

**Authors:** Dragos-Gabriel Iancu, Razvan Gheorghita Mares, Liviu Cristescu, Radu-Adrian Suteu, Andreea Varga, Ioan Tilea

**Affiliations:** 1 Doctoral School, George Emil Palade University of Medicine, Pharmacy, Science, and Technology of Targu Mures, 540142 Targu Mures, Romania; dragos-gabriel.iancu@umfst.ro (D.-G.I.); liviu.cristescu@umfst.ro (L.C.); 2 Faculty of Medicine, George Emil Palade University of Medicine, Pharmacy, Science, and Technology of Targu Mures, 540142 Targu Mures, Romania; ioan.tilea@umfst.ro; 3 Department of Cardiology I, The Emergency Institute for Cardiovascular Diseases and Transplantation, 540136 Targu Mures, Romania; radu.suteu@umfst.ro; 4 Faculty of Medicine in English, George Emil Palade University of Medicine, Pharmacy, Science, and Technology of Targu Mures, 540142 Targu Mures, Romania; andreea.varga@umfst.ro

**Keywords:** pulmonary arterial hypertension, chronic thromboembolic pulmonary hypertension, inflammation-based biomarkers, neutrophil-to-lymphocyte ratio, platelet-to-lymphocyte ratio, neutrophil-percentage-to-albumin ratio, lymphocyte-to-monocyte ratio, systemic immune-inflammation index, length of hospital stay, mortality assessment

## Abstract

Pulmonary arterial hypertension (PAH) and chronic thromboembolic pulmonary hypertension (CTEPH) are characterized by high morbidity and mortality. We retrospectively analyzed 468 hospital admissions from 80 patients to evaluate the prognostic value of inflammation-based hematologic indices, including the neutrophil-to-lymphocyte ratio (NLR), platelet-to-lymphocyte ratio (PLR), neutrophil-percentage-to-albumin ratio (NPAR), lymphocyte-to-monocyte ratio (LMR), and systemic immune-inflammation index (SII). All biomarker–outcome associations were specified a priori as exploratory in this hypothesis-generating design. In PAH, both NPAR and SII were associated with in-hospital mortality (odds ratio [OR] 1.129, 95% confidence interval [CI] 1.011–1.261, *p* = 0.031; OR 1.001, 95% CI 1.000–1.002, *p* = 0.002), post-discharge mortality (NPAR OR 1.181, 95% CI 1.062–1.313, *p* = 0.002), and poorer overall survival (log-rank *p* = 0.002 and *p* = 0.012, respectively). Higher LMR was associated with reduced in-hospital mortality (OR 0.291, 95% CI 0.108–0.790, *p* = 0.015), while NLR predicted increased in-hospital mortality. In CTEPH, NLR and LMR were the strongest predictors, correlating with worse survival (log-rank *p* = 0.007 and *p* = 0.044) and higher post-discharge mortality (NLR OR 1.289, 95% CI 1.029–1.615, *p* = 0.027). Receiver operating characteristic (ROC) analysis suggests the potential value of SII in PAH and the promising performance of NPAR in CTEPH. Inflammation-based hematologic indices, particularly NPAR, SII, and NLR, may provide valuable prognostic information and may serve as practical, non-invasive tools for predicting hospitalization duration and mortality in PAH and CTEPH.

## 1. Introduction

Pulmonary hypertension (PH) is a progressive and heterogeneous disorder defined by elevated pulmonary vascular resistance (PVR) and right ventricular dysfunction [1]. Despite advances in diagnosis and treatment, PH remains associated with substantial morbidity, prolonged hospitalization, and mortality, highlighting the need for the early and accurate prediction of these outcomes [2].

In pulmonary arterial hypertension (PAH) and chronic thromboembolic pulmonary hypertension (CTEPH), risk assessment is currently based on symptom severity, hemodynamic measurements, and natriuretic peptides. In CTEPH, persistent organized thrombi after pulmonary embolism, together with a secondary small-vessel “microvasculopathy” sustained by inflammatory and pro-coagulant pathways, are key drivers of disease progression and adverse outcomes. These pathophysiologic insights support growing interest in inflammation-based biomarkers that may refine prognostic evaluation and inform clinical decision-making [3,4,5].

Inflammation-based hematologic indices derived from routine blood tests have emerged as reproducible indicators of systemic immune activation. The neutrophil-to-lymphocyte ratio (NLR), platelet-to-lymphocyte ratio (PLR), neutrophil-percentage-to-albumin ratio (NPAR), lymphocyte-to-monocyte ratio (LMR), and systemic immune-inflammation index (SII) are increasingly recognized for their prognostic significance across cardiovascular conditions.

Elevated NLR has been linked to adverse outcomes and increased mortality in coronary artery disease (CAD) and chronic left heart failure (HF), while its prognostic relevance in precapillary PH has been poorly investigated [6,7]. Available evidence suggests that higher NLR values are associated with greater functional impairment and increased mortality risk [8].

PLR has been proposed as an indicator of subclinical inflammation across various conditions with elevated PLR correlating with unfavorable outcomes in acute HF [9].

The combined use of NLR and PLR has been shown to predict post-discharge cardiac death in patients with acute HF and preserved ejection fraction [10].

NPAR, which relates neutrophil percentage to plasma albumin, has been validated as a mortality predictor in acute myocardial infarction, cardiogenic shock, CAD, and HF in critical care settings [11,12,13,14].

Lymphocytes and monocytes are central to both innate and adaptive immunity, and the LMR reflects the balance between immune activation and suppression [15,16].

Elevated monocyte counts indicate the progression of immune-mediated processes, whereas reduced circulating lymphocytes suggest their migration to sites of inflammation [17].

The integration of these parameters into a single index—LMR, provides prognostic insight [18]. Higher LMR values are generally associated with a more balanced immune response, improved survival, and shorter hospitalizations in cardiovascular disease [19,20]. In contrast, lower LMR, typically driven by increased monocyte counts, corresponds to persistent inflammation, vascular remodeling, and adverse outcomes.

Compared with these indices, the role of inflammation in PAH and CTEPH has been less well-characterized. SII, integrating platelet, neutrophil, and lymphocyte counts, has been associated with PH progression, and with increased mortality in acute pulmonary embolism [21,22,23].

Although substantial progress has been achieved through updated ESC/ERS guidelines and modern risk assessment strategies, outcomes in PH remain unsatisfactory. Approaches centered on clinical symptoms and hemodynamic measurements often fail to identify high-risk patients early in the disease course. Inflammation-based hematologic indices derived from routine laboratory tests provide inexpensive, non-invasive tools with the potential to predict length of hospital stay (LOS) and mortality.

The present study explored and aimed to generate a new working hypothesis in research regarding the prognostic value of the NLR, PLR, NPAR, LMR, and SII in patients with PAH and CTEPH, with specific focus on LOS, in-hospital mortality, and post-discharge mortality. While NLR and PLR have been more frequently examined in PH, data on NPAR and SII are scarce, and no study to date has comprehensively evaluated their prognostic significance in both PAH and CTEPH. This study therefore addresses an important knowledge gap by investigating the prognostic relevance of these underexplored indices.

## 2. Results

### 2.1. Baseline Characteristics of PAH and CTEPH Patients

A total of 80 patients were included in the analysis: 54 (67.5%) with PAH and 26 (32.5%) with CTEPH. Female predominance was evident in the PAH group, whereas the sex distribution in CTEPH was more balanced. Median age was significantly lower in PAH compared with CTEPH. In both cohorts, most patients exhibited moderate-to-severe functional limitation, with the majority classified as World Health Organization functional class (WHO-FC) II or III. Detailed baseline demographic, hemodynamic, and comorbidity profiles are provided in Table 1.

### 2.2. Inflammatory Laboratory Markers in PAH and CTEPH

Inflammation-related laboratory parameters and biomarker indices are presented in Table 2. Although most hematologic parameters showed no significant differences between groups, patients with CTEPH had significantly higher PLR and SII values and lower LMR, indicating a distinct inflammatory profile compared with PAH.

### 2.3. Length of Hospital Stay (LOS)

In the PAH cohort, LOS demonstrated weak but statistically significant positive correlations with NLR, PLR, NPAR, and SII, whereas LMR was inversely correlated.

In the CTEPH cohort, stronger correlations were observed for NPAR, NLR, and LMR, indicating a more pronounced relationship between the inflammatory indices and hospitalization. LMR was inversely correlated with LOS, supporting a potential protective effect, while SII showed a weaker yet significant association. PLR was not significantly correlated with LOS in CTEPH patients.

Correlation coefficients between inflammation-based hematologic indices and LOS are presented in Table 3.

LOS exhibited non-normality and heteroscedasticity on diagnostic testing; accordingly, we modeled LOS with a Gamma generalized linear model (log link). Adjusting for diagnostic group (PAH vs. CTEPH), NLR was positively associated with LOS (β = 0.08296 ± 0.01591, *p* = 2.8 × 10^−7^), corresponding to an 8.6% (95% CI 5.3–12.1) longer stay per 1-unit higher NLR. Findings were directionally consistent in sensitivity analyses using log(LOS) linear models and ordinary least squares (OLS) with heteroskedasticity-consistent variant 3 (HC3) robust standard errors.

After adjustment for demographics, comorbidities, and PH-specific therapies, distinct patterns emerged across cohorts: in PAH, NLR was the only inflammatory marker associated in adjusted models with LOS (F = 7.69, *p* = 0.006, partial η^2^ = 0.027), while PLR, NPAR, LMR, and SII showed no significant effects. In contrast, in CTEPH, systemic inflammation was a consistent predictor of prolonged hospitalization, with NLR (F = 10.83, *p* = 0.001, partial η^2^ = 0.055), PLR (F = 11.37, *p* = 0.001, partial η^2^ = 0.058) and LMR (F = 19.27, *p* < 0.001, partial η^2^ = 0.094) all showing associations in adjusted models, whereas NPAR and SII did not, the latter showing only a weak trend (*p* = 0.081). These ANCOVA/OLS results are secondary to the primary Gamma log-link analysis and should be interpreted cautiously given heteroscedasticity and within-patient clustering.

### 2.4. Biomarkers as Predictors of In-Hospital Mortality

Univariate logistic regression analysis was performed to evaluate the prognostic value of the indices for in-hospital mortality (Table 4).

In the PAH cohort, several indices were significantly associated with adverse outcomes. Elevated NLR was strongly predictive (OR = 1.582, *p* < 0.001), with each unit increase corresponding to a 58.2% higher risk of in-hospital death. Both NPAR (OR = 1.129, *p* = 0.031) and SII (OR = 1.001, *p* = 0.002) were also associated with increased mortality, whereas LMR demonstrated a significant inverse association (OR = 0.291, *p* = 0.015), suggesting a protective effect.

In the CTEPH cohort, none of the indices reached statistical significance. Although LMR showed a relatively low odds ratio (OR = 0.227), the wide confidence interval and borderline significance (*p* = 0.089) indicate that the association was not robust.

These findings indicate that inflammation-based hematologic indices have stronger prognostic value for in-hospital mortality in PAH compared with CTEPH, with LMR showing a potential protective association.

Multivariable analysis adjusting for demographic characteristics, comorbid conditions, and PH-specific treatments identified several inflammatory markers that were associated in adjusted models with in-hospital mortality. In the PAH cohort, NLR (OR = 1.48, 95% CI 1.13–1.93; *p* = 0.005), NPAR (OR = 1.23, 95% CI 1.04–1.46; *p* = 0.013), and SII (OR = 1.01, 95% CI 1.001–1.005; *p* = 0.016) were positively associated with mortality risk, whereas a lower LMR was also predictive (OR = 0.23, 95% CI 0.07–0.80; *p* = 0.021). PLR did not demonstrate an independent effect. In the CTEPH cohort, NLR (OR = 1.81, 95% CI 1.01–3.25; *p* = 0.045), PLR (OR = 1.02, 95% CI 1.00–1.04; *p* = 0.043), and SII (OR = 1.02, 95% CI 1.00–1.09; *p* = 0.040) retained prognostic significance, while LMR showed only a borderline trend (OR = 0.01, 95% CI 0.00–1.28; *p* = 0.064) and NPAR was not predictive. This apparent shift from univariate to multivariable significance likely reflects the reduction in confounding by age, sex, comorbidity burden, and PH-specific therapies. Such adjustment helps isolate the independent contribution of systemic inflammation to in-hospital mortality, particularly in heterogeneous cohorts with a limited number of events.

**Figure 1:**
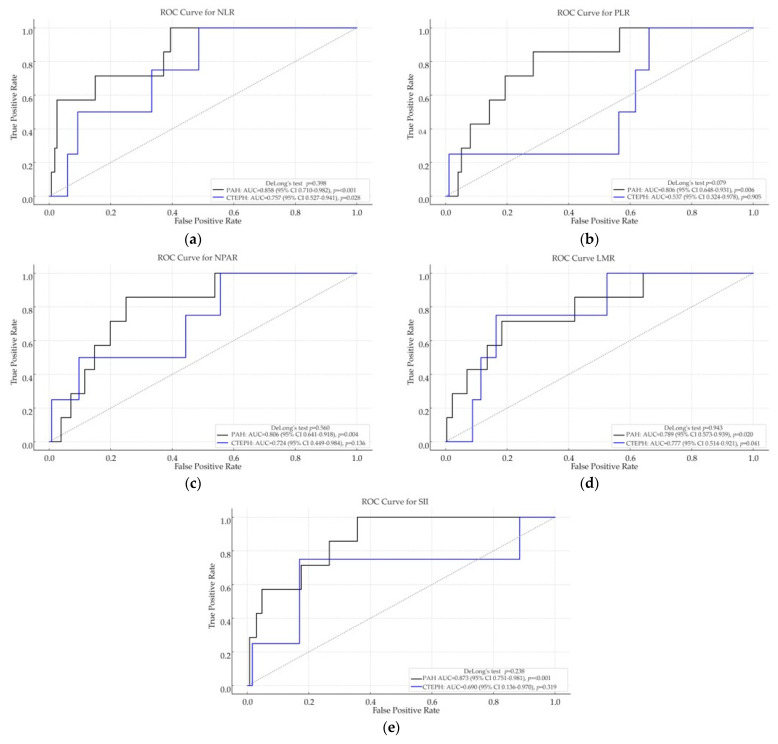
Receiver operating characteristic (ROC) curve analyses of inflammation-based hematologic indices for predicting in-hospital mortality in patients with pulmonary arterial hypertension (PAH) and chronic thromboembolic pulmonary hypertension (CTEPH). Panels display: (**a**) neutrophil-to-lymphocyte ratio (NLR); (**b**) platelet-to-lymphocyte ratio (PLR); (**c**) neutrophil-percentage-to-albumin ratio (NPAR); (**d**) lymphocyte-to-monocyte ratio (LMR); and (**e**) systemic immune-inflammation index (SII). Indices with higher area under the curve (AUC) indicate stronger discriminatory performance for in-hospital mortality.

Receiver operating characteristic (ROC) curve analyses were performed to evaluate the discriminative ability of each inflammatory index for in-hospital mortality in both the PAH and CTEPH cohorts (see Figure 1).

Among patients with PAH, all five inflammation-based indices demonstrated strong discriminatory performance for in-hospital mortality. The highest AUC was observed for SII (0.873, *p* < 0.001), followed by NLR (0.858, *p* < 0.001), PLR (0.806, *p* = 0.006), NPAR (0.806, *p* = 0.004), and LMR (0.789, *p* = 0.020).

In the CTEPH cohort, overall discriminative performance was lower. The highest AUCs were recorded for LMR (0.777, *p* = 0.041) and NLR (0.757, *p* = 0.028); however, pairwise comparisons using DeLong’s test did not reveal significant differences (e.g., *p* = 0.943 for LMR, *p* = 0.398 for NLR). PLR, SII, and NPAR were not statistically significant predictors in this cohort.

Notably, while univariate logistic regression did not identify significant associations in CTEPH, both LMR and NLR achieved significant discriminatory capacity in ROC analysis. This discrepancy may reflect the ability of ROC methodology to capture nonlinear or distribution-dependent patterns that are not adequately represented by the assumptions of logistic regression.

These results indicate that inflammation-based hematologic indices—particularly SII, NLR, and LMR—may be valuable prognostic tools for in-hospital mortality in PAH, whereas their predictive accuracy in CTEPH appears more limited.

Across the PAH and CTEPH cohorts, DeLong pairwise comparisons of the ROC curves showed no significant differences (*p* > 0.05).

### 2.5. Hematologic Indices as Predictors of Post-Discharge Mortality

In the PAH cohort, both NPAR and LMR were significantly associated with adverse outcomes in univariate analyses (NPAR: OR = 1.181, *p* = 0.002; LMR: OR = 0.291, *p* = 0.015), indicating that higher NPAR and lower LMR values predicted increased post-discharge mortality. In the CTEPH cohort, elevated NLR was significantly associated with post-discharge mortality (OR = 1.289, *p* = 0.027), supporting the prognostic relevance of neutrophil-driven inflammation in long-term outcomes. No other hematologic indices demonstrated significant associations. Taken together, these results suggest distinct prognostic profiles between PAH and CTEPH, with NPAR emerging as a predictor of post-discharge mortality in PAH, whereas NLR provides prognostic value in CTEPH (Table 5).

In the PAH cohort, after adjustment for demographics, comorbidities, and PH-specific therapies, NPAR remained associated with post-discharge mortality (OR = 1.17, 95% CI: 1.05–1.31; *p* = 0.004). None of the other variables retained prognostic significance after adjustment. In contrast, in the CTEPH cohort, multivariable sensitivity analyses indicated that none of the inflammatory indices (NLR, PLR, NPAR, LMR, or SII) remained significantly associated with post-discharge mortality once potential confounders were accounted for.

In CTEPH, the low event counts (post-discharge mortality 3/187) led to non-estimable Firth logistic models for all biomarkers, even under biomarker-only specifications. Thus, multivariable inference is underpowered, and the findings are reported exploratorily, without independence claims.

**Figure 2:**
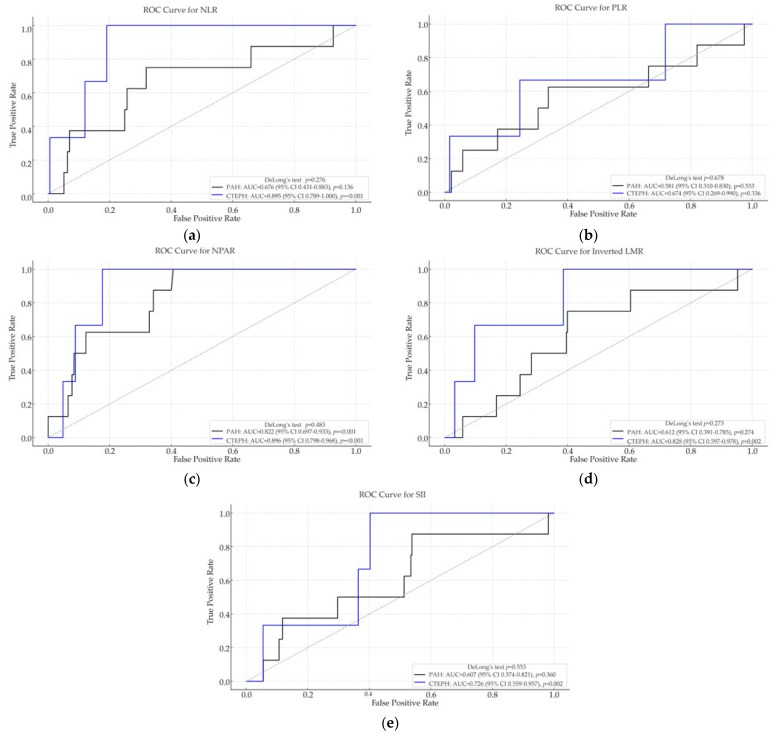
Receiver operating characteristic (ROC) curve analyses of inflammation-based hematologic indices for predicting post-discharge mortality in patients with pulmonary arterial hypertension (PAH) and chronic thromboembolic pulmonary hypertension (CTEPH). Panels show: (**a**) neutrophil-to-lymphocyte ratio (NLR); (**b**) platelet-to-lymphocyte ratio (PLR); (**c**) neutrophil-percentage-to-albumin ratio (NPAR); (**d**) lymphocyte-to-monocyte ratio (LMR); and (**e**) systemic immune-inflammation index (SII). Higher area under the curve (AUC) values reflects greater discriminatory ability for post-discharge mortality risk.

ROC curve analysis was conducted to evaluate the discriminative ability of inflammation-based biomarkers for predicting post-discharge mortality in PAH and CTEPH patients (Figure 2).

In the PAH cohort, most biomarkers demonstrated modest predictive performance, with NPAR showing very good discrimination (AUC = 0.822, *p* < 0.001), while NLR (AUC = 0.676, *p* = 0.136) and inverted LMR (AUC = 0.612, *p* = 0.274) did not reach statistical significance. PLR and SII showed poor prognostic value, with AUCs close to 0.5 and non-significant *p*-values, suggesting limited utility in predicting post-discharge outcomes in PAH.

In contrast, in the CTEPH group, several biomarkers demonstrated significantly better ROC performance. NPAR (AUC = 0.896, *p* < 0.001) and NLR (AUC = 0.895, *p* < 0.001) showed excellent discrimination, while inverted LMR (AUC = 0.828, *p* = 0.002) and SII (AUC = 0.726, *p* = 0.002) also exhibited good predictive ability. These findings suggest that inflammatory biomarkers may be more relevant for post-discharge mortality in CTEPH compared with PAH.

These results indicate that NLR, NPAR, and LMR are the most promising indices for predicting post-discharge mortality, with prognostic relevance particularly evident in the CTEPH cohort.

In PAH, NPAR achieved the highest AUC and significantly outperformed LMR (ΔAUC = 0.210; 95% CI 0.030–0.418; *p* = 0.022), while all other pairwise differences were non-significant. In CTEPH, NLR discriminated better than SII (ΔAUC = 0.169; 95% CI 0.043–0.294; *p* = 0.003), with no other pairwise comparisons reaching significance.

### 2.6. Survival Analysis According to Inflammation-Based Hematologic Indices

**Figure 3:**
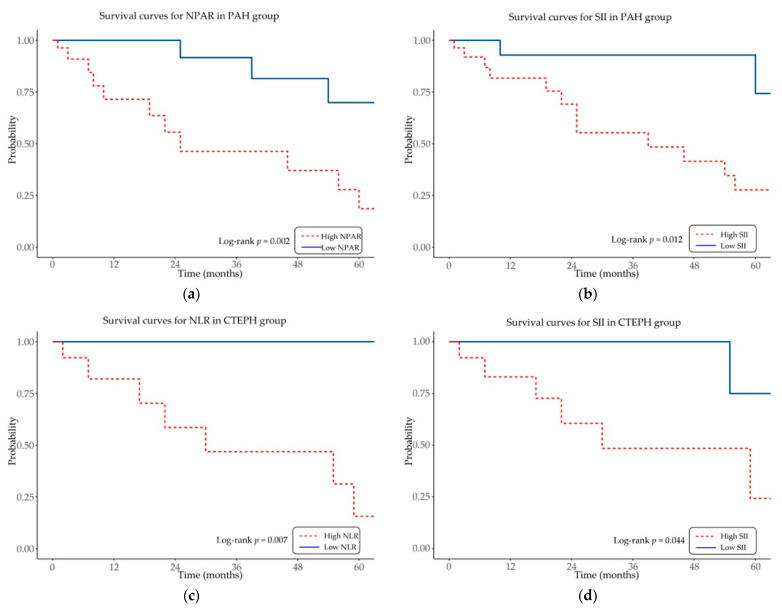
Kaplan–Meier survival curves showing overall survival according to inflammation-based hematologic indices in patients with pulmonary arterial hypertension (PAH) and chronic thromboembolic pulmonary hypertension (CTEPH). Panels display: (**a**) neutrophil-percentage-to-albumin ratio (NPAR) in PAH; (**b**) systemic immune-inflammation index (SII) in PAH; (**c**) neutrophil-to-lymphocyte ratio (NLR) in CTEPH; and (**d**) SII in CTEPH. In both cohorts, elevated index values were associated with reduced overall survival compared with lower values.

Kaplan–Meier survival analysis was conducted to assess the prognostic impact of inflammation-based hematologic indices on overall survival in patients with PAH and CTEPH (Figure 3).

In the PAH cohort, elevated NPAR and SII were significantly associated with reduced overall survival (log-rank *p* = 0.002 and *p* = 0.012, respectively). In the CTEPH cohort, higher NLR and SII values were similarly linked to decreased overall survival (log-rank *p* = 0.007 and *p* = 0.044, respectively).

Other indices, including PLR and LMR, did not show significant associations with survival in either cohort (log-rank *p* > 0.05).

These results indicate that selected inflammation-based hematologic indices, particularly NPAR, SII, and NLR, provide prognostic information for mortality in specific PH subgroups.

This study explored the prognostic value of inflammation-based hematologic indices in patients with PAH and CTEPH. In keeping with the exploratory, hypothesis-generating design, the findings—limited by modest event numbers, particularly in the CTEPH cohort, and absence of clustering adjustment—require confirmation in larger, multicenter cohorts. Nonetheless, the observed associations suggest that such readily available biomarkers may hold clinical relevance, warranting further validation in broader patient populations.

## 3. Discussion

This retrospective observational study investigated the prognostic relevance of inflammation-based hematologic indices (NLR, PLR, NPAR, LMR, and SII) in patients with PAH and CTEPH. The predictive significance of these biomarkers differed across disease subtypes and clinical endpoints. In the PAH cohort, NPAR and SII were associated in adjusted models with in-hospital mortality, whereas NPAR also predicted post-discharge mortality. Additionally, NLR was the only marker associated with LOS after adjustment. In the CTEPH cohort, LOS was associated with NLR, PLR, and LMR, while in-hospital mortality was predicted by NLR, PLR, and SII. None of the indices retained prognostic significance for post-discharge mortality in this group. Although the effect sizes for LOS were moderate, the findings consistently support a role of systemic inflammation in determining hospitalization burden in PH.

To align biomarkers with disease biology, we interpreted NLR/PLR/SII as proxies for thrombo-inflammatory activation and endothelial injury, LMR for monocyte–lymphocyte balance, and NPAR as an integrated inflammatory/albumin signal; in CTEPH, these profiles likely mirror chronic thrombus organization and microvascular remodeling rather than exerting causal effects.

These results underscore the heterogeneity of inflammatory mechanisms between PAH and CTEPH and demonstrate the potential utility of readily available hematologic indices for refined short-term prognostication. A novel contribution of this study lies in the evaluation of NPAR and SII—composite indices integrating neutrophil activity, serum albumin as a negative acute-phase reactant, and the platelet–neutrophil–lymphocyte interaction. Their prognostic significance in PAH and CTEPH has not been systematically characterized previously, thereby highlighting the originality and potential clinical relevance of these findings.

As complementary descriptive and visual tools, ROC/AUC, and Kaplan–Meier curves were retained to summarize discrimination and crude risk separation. Importantly, in tiny-event settings like CTEPH, ROC-based discrimination may appear acceptable even when adjusted regression becomes unstable (e.g., wide, or non-estimable coefficients). Therefore, discriminative accuracy without stable adjusted effect estimates should be interpreted with caution, and our CTEPH signals are considered exploratory pending validation.

### 3.1. Neutrophil-to-Lymphocyte Ratio (NLR)

The neutrophil-to-lymphocyte ratio (NLR) is an established index of systemic inflammation and endothelial dysfunction and has been linked to adverse outcomes in pulmonary hypertension (PH), including hospitalization and mortality [24]. A threshold of 4.14, identified through ROC analysis, has previously been shown to predict survival in Kaplan–Meier analyses [25]. In our cohort, median NLR values were 3.03 in PAH and 3.01 in CTEPH, indicating a moderate inflammatory burden in both subgroups.

In both cohorts, NLR was consistently associated with adverse clinical outcomes. In PAH, elevated NLR strongly predicted in-hospital mortality and demonstrated good discriminatory accuracy, although it did not retain prognostic value for long-term outcomes. In CTEPH, higher NLR values correlated with reduced overall survival and increased post-discharge mortality in univariate and ROC analyses. However, after multivariable adjustment, NLR remained associated with LOS in adjusted analyses in both PAH and CTEPH, and of in-hospital mortality in both subgroups. Its association with post-discharge mortality in CTEPH was no longer significant after adjustment, suggesting that this relationship may be partly explained by confounding factors. Previous studies similarly reported that higher NLR values were associated with advanced functional class and increased mortality, with patients presenting NLR ≥ 2.62 exhibiting reduced five-year survival [26].

In CTEPH, increased NLR correlated with reduced overall survival and higher post-discharge mortality, consistent with prior evidence supporting its prognostic utility in patients undergoing pulmonary endarterectomy [24]. Yogeswaran et al. further demonstrated that NLR was a reliable prognostic marker both at the baseline and during follow-up, with predictive performance comparable to the European Society of Cardiology/European Respiratory Society (ESC/ERS) risk stratification scheme [27].

### 3.2. Platelet-to-Lymphocyte Ratio (PLR)

PLR demonstrated limited prognostic relevance in the PAH cohort, where it did not retain prognostic significance with clinical outcomes after adjustment. In contrast, in the CTEPH cohort, PLR remained associated in adjusted models with both LOS and in-hospital mortality, suggesting that platelet-driven inflammatory mechanisms may exert a more prominent role in this subgroup.

These findings align with previous reports describing heterogeneous and sometimes inconsistent associations between PLR and mortality across large patient cohorts with heart failure [refs], supporting the notion that the prognostic impact of PLR may vary depending on disease phenotype and inflammatory context [28].

### 3.3. Neutrophil-Percentage-to-Albumin Ratio (NPAR)

In the PAH cohort, NPAR emerged as a robust prognostic marker, remaining associated with both in-hospital and post-discharge mortality, while showing no association with LOS. In the CTEPH cohort, NPAR correlated with LOS and demonstrated excellent discriminatory accuracy for post-discharge mortality in ROC analysis; however, these associations did not persist in multivariable models, suggesting that its predictive value may be influenced by confounding factors or by nonlinear relationships more effectively captured through ROC-based evaluation.

Evidence on the role of NPAR in PH is scarce; however, elevated NPAR has been linked to increased systemic inflammation and to higher short- and long-term mortality in patients with acute myocardial infarction, sepsis, and cardiogenic shock [11,29,30]. The present findings suggest that NPAR may serve as a robust and readily available prognostic index in both PAH and CTEPH, warranting confirmation in prospective studies.

### 3.4. Lymphocyte-to-Monocyte Ratio (LMR)

Across both cohorts, LMR was inversely associated with hospitalization burden, indicating that lower values—reflecting monocyte-driven inflammation and relative lymphocyte depletion—were linked to prolonged LOS and adverse outcomes, consistent with previous reports [31,32]. In the PAH cohort, reduced LMR was significantly associated with both in-hospital and post-discharge mortality in univariate and ROC analyses; however, after multivariable adjustment, it remained predictive only of in-hospital mortality, with modest discriminatory accuracy. In the CTEPH cohort, associations followed a similar pattern, and LMR retained an independent inverse relationship with LOS, further supporting its role as a marker of systemic inflammation and disease severity in PH.

LMR has been indicated as an important marker of immune regulation and systemic inflammation in PH progression, reflecting lymphocyte preservation and monocyte-driven inflammation [33].

Prior research in patients with acute coronary syndromes identified an LMR threshold of 3.00, below which one-year mortality was significantly increased [34]. Similarly, in acute pulmonary embolism, non-survivors exhibited markedly lower LMR values, supporting its utility as a marker of adverse outcomes in thromboembolic disease [35].

### 3.5. Systemic Immune-Inflammation Index (SII)

The systemic immune-inflammation index (SII) has been extensively evaluated in cardiovascular disease, yet its role in PH remains insufficiently characterized. By integrating platelet, neutrophil, and lymphocyte counts into a single composite measure, SII reflects the interplay between platelet activity, innate inflammation, and adaptive immune suppression [21].

Elevated SII has consistently been associated with adverse in-hospital and long-term outcomes across cardiovascular and respiratory diseases and has further been linked to PH progression and increased mortality in acute pulmonary embolism [23,36,37,38,39,40].

In the PAH cohort, higher SII values were significantly associated with prolonged LOS and exhibited strong predictive accuracy for in-hospital mortality, while demonstrating moderate prognostic utility for long-term survival. In the CTEPH cohort, elevated SII showed comparable associations with LOS and mortality in univariate and ROC analyses, although the effect sizes were less pronounced. Following multivariable adjustment, SII no longer remained an independent predictor of LOS—showing only a weak trend in CTEPH—but retained independent prognostic significance for in-hospital mortality in both cohorts. These findings support the role of SII as a composite marker of systemic inflammation and confirm its relevance for short-term risk stratification in PH.

The apparent discrepancies between ROC/Kaplan–Meier and multivariable regression analyses are expected, as these methods capture different dimensions of prognostic performance. ROC and Kaplan–Meier analyses can reflect nonlinear relationships, distribution-dependent effects, and group-level survival differences, whereas regression models are more sensitive to confounding and collinearity. As a result, associations such as those observed for NPAR and NLR with post-discharge mortality in CTEPH may attenuate after adjustment without invalidating the univariate or ROC findings. These results should not be regarded as contradictory but rather as complementary, emphasizing the need for robust modeling in larger cohorts and the careful interpretation of biomarker performance across multiple analytical approaches.

Established risk models in pulmonary hypertension—such as REVEAL and the ESC/ERS 4-strata risk assessment model—provide multiparametric estimates of prognosis, whereas the inflammatory indices evaluated in this study (NLR, PLR, NPAR, LMR, and SII) reflect systemic immune–inflammatory balance, a dimension not explicitly captured by current tools. These indices may complement guideline-based stratification by identifying patients with disproportionate inflammatory activation who could benefit from closer follow-up, earlier reassessment, repeated biomarker testing, or lower thresholds for treatment escalation or referral. However, they should not replace established prognostic frameworks. Given the single-center, retrospective, admission-level design and data-driven cut-offs, the present findings are exploratory. Future studies should apply cluster-aware analytical methods and include external multicenter validation, prespecified thresholds, calibration assessment, and decision-curve analysis to determine clinical utility. If validated, inflammatory indices could be pragmatically integrated into multimodal risk assessment—alongside established instruments such as REVEAL and the ESC/ERS 4-strata risk assessment model—to support more individualized patient management.

### 3.6. Limitations and Future Directions

While this study provides valuable insights into the prognostic relevance of hematologic and inflammation-based biomarkers in PAH and CTEPH, several limitations should be acknowledged. First, its retrospective, single-center design may introduce selection bias and restrict the generalizability of results. Second, although the sample size of 468 hospital admissions is comparable to previous reports, the number of events per variable remains limited, which may affect the robustness of multivariable models and preclude detailed stratification by PH etiology, functional class, or specific therapies.

Although PAH and CTEPH differ in pathogenesis, they were analyzed separately to highlight both shared and distinct inflammatory patterns, rather than for direct comparison.

The analysis was conducted at the level of hospital admissions, with some patients contributing multiple observations. As no statistical adjustment for intra-patient clustering was applied, standard errors may have been underestimated, and results should be regarded as exploratory. Despite multivariable adjustment, residual confounding cannot be excluded, and the limited number of events per variable restricted the power of some models. Future studies should validate these findings in larger, prospective, multicenter cohorts using mixed-effects or generalized estimating equations (GEEs) models and incorporating nonlinear specifications to provide more robust estimates.

Biomarker levels were assessed at each hospital admission, allowing for repeated cross-sectional evaluations. However, the lack of structured longitudinal follow-up limits our ability to assess biomarker trajectories over time or their dynamic response to therapeutic interventions.

Furthermore, comorbidity data were extracted from discharge documentation and diagnostic coding, which may vary between clinicians and across admissions. Such heterogeneity could have led to an underestimation or misclassification of certain conditions, including obstructive sleep apnea, as previously reported in similar single-center retrospective studies [41]. Comorbidities were based on clinician-verified discharge diagnoses and medication lists but lacked standardized severity indices; therefore, residual confounding (e.g., by hypertension or coronary heart disease) cannot be excluded. This limitation may be more pronounced in CTEPH, where event counts were low and models necessarily parsimonious. These factors emphasize the exploratory nature of our findings and highlight the need for validation in larger cohorts using standardized comorbidity measures.

Future registry-based work should use dedicated, disease-specific questionnaires and harmonized definitions, complemented by audit and continuing medical education in coding practices, to reduce variability and improve comorbidity ascertainment.

Furthermore, the absence of an external validation cohort restricts the generalizability of our findings. These results should thus be framed as hypothesis-generating and require confirmation in larger, prospective, multicenter studies that include diverse patient populations.

Standardized cut-off values for biomarkers such as NLR, PLR, NPAR, LMR, and SII remain to be established in PH, and our proposed thresholds require further validation. Moreover, we focused primarily on inflammatory and hematologic indices; the addition of emerging markers of endothelial dysfunction (e.g., endothelin-1, von Willebrand factor) or systemic inflammation (e.g., interleukin-6, C-reactive protein) may enhance prognostic stratification.

Importantly, the systematic evaluation of NPAR and SII in PAH and CTEPH represents a novel contribution, as these indices have not been previously validated in this setting. By highlighting their independent associations with clinically relevant outcomes, our study provides incremental value beyond earlier reports that primarily focused on NLR, PLR, and LMR.

Analyses were conducted at the admissions level, with some patients contributing multiple observations. Because we did not apply cluster-aware estimators in this single-center dataset, within-patient correlation may have underestimated standard errors and could affect model stability, particularly in the CTEPH subgroup where events were very few. In larger cohorts, appropriate approaches—such as mixed-effects models with patient-level random intercepts, cluster-robust (patient-level) standard errors or GEEs—should be used to account for the clustered structure, quantify intra-patient correlation, and provide more reliable uncertainty estimates.

This study was not powered to demonstrate incremental prognostic value of inflammation-based indices beyond guideline-recommended multiparametric tools (e.g., REVEAL score, ESC/ERS 4-strata risk assessment model) and should be viewed as hypothesis-generating. Establishing clinical relevance will require the formal testing of added predictive value using (a) likelihood-ratio tests against base models with standard predictors, (b) changes in discrimination (ΔAUC/c-index with DeLong 95% CIs), (c) calibration (intercept/slope and calibration plots), (d) reclassification (IDI/NRI with confidence intervals), and (e) decision-curve analysis to assess net clinical benefit. Such evaluations—ideally with prespecified cut-offs, external validation, and serial measurements—are necessary before considering routine implementation as adjuncts to existing scores.

Future research should focus on prospective, multicenter studies with larger, more diverse PH populations to validate these findings. Longitudinal designs incorporating repeated biomarker assessments are needed to determine whether dynamic changes in indices add incremental prognostic value beyond baseline levels. Integration of inflammation-based hematologic indices with hemodynamic, imaging, and molecular biomarkers could improve risk stratification models. Additionally, advanced analytic approaches, including machine-learning algorithms, may help construct multi-parameter predictive tools with higher accuracy and clinical applicability. Finally, formal cost-effectiveness analyses are required to establish the clinical utility of these indices in routine practice.

## 4. Materials and Methods

### 4.1. Study Design and Population

This study was designed as a hypothesis-generating, retrospective, observational analysis based on data from a single-center PH registry. The dataset comprised 468 hospital admissions from 80 unique patients with PAH or CTEPH, recorded between September 2015 and October 2024 at the PH Center, Department of Internal Medicine II–Cardiology, County Emergency Clinical Hospital, Targu Mures, Romania.

Patients were classified into PH subgroups according to the 2015 and 2022 ESC/ERS guidelines. The study cohort included individuals with pulmonary arterial hypertension (PAH, Group 1) and chronic thromboembolic pulmonary hypertension (CTEPH, Group 4). PAH subtypes included idiopathic PAH (IPAH, Group 1.1), PAH associated with congenital heart disease (CHD-PAH, Group 1.4.4), and PAH related to connective tissue disease (CTD-PAH, Group 1.4.1). CTEPH (Group 4.1) was defined as persistent pulmonary vascular obstruction following at least one episode of acute pulmonary embolism.

### 4.2. Inclusion and Exclusion Criteria

Standardized inclusion and exclusion criteria were applied to ensure diagnostic consistency and reliable data collection. Eligible participants were adults aged 18 years or older with a confirmed diagnosis of PAH or CTEPH, established in accordance with the 2015 and 2022 ESC/ERS guidelines. Hospitalizations lasting more than 48 h were included, irrespective of whether the admission was prompted by acute decompensation, scheduled clinical reassessment, or therapeutic escalation. Only cases with complete biochemical data enabling the calculation of inflammation-based hematologic indices (NLR, PLR, NPAR, LMR, and SII) were eligible for analysis.

Admissions were excluded if laboratory data were incomplete, follow-up information was unavailable, or an active infection (viral, bacterial, or fungal) was present at the time of admission. Patients experiencing a documented flare of systemic autoimmune or inflammatory disease (such as systemic lupus erythematosus or rheumatoid arthritis) requiring initiation or escalation of immunosuppressive therapy were also excluded.

Patients with stable connective tissue disease-associated PAH (Group 1.4.1) receiving maintenance therapy, without evidence of active flare at admission, were eligible for inclusion.

### 4.3. Treatment, Management, and Disease Severity Parameters

Management was conducted in accordance with contemporary ESC/ERS guidelines for PH, and treatment protocols were applied uniformly across the cohort to ensure consistent therapeutic exposure.

Disease severity was evaluated using the six-minute walk distance (6MWD) and WHO-FC.

### 4.4. Laboratory Assessments

Blood samples were obtained within 2 h of admission, following a fasting period of at least 8 h. White blood cell (WBC), lymphocyte, granulocyte (neutrophil), and monocyte counts were measured using a Sysmex XN-550 analyzer (Sysmex Corporation, Kobe, Japan). Biochemical analyses were performed on a Konelab Prime 60i system (Thermo Fisher Scientific Inc., Waltham, MA, USA). All measurements were performed in an ISO 15189-certified laboratory (International Organization for Standardization) [42,43].

### 4.5. Inflammatory and Biochemical Markers

The NLR was calculated as the neutrophil count divided by the lymphocyte count; the PLR as the platelet count divided by the lymphocyte count; the NPAR as the neutrophil percentage divided by plasma albumin; and the LMR as the lymphocyte count divided by the monocyte count. The SII was determined as (neutrophil count × platelet count)/lymphocyte count. All values were derived from routine complete blood count and biochemical analyses performed at admission. Temporal variability in biomarker levels was not assessed; all analyses were based on measurements obtained at index admission.

### 4.6. Comorbidities

Comorbidities were systematically documented across major categories—cardiovascular (hypertension, coronary artery disease, atrial fibrillation, deep vein thrombosis), metabolic (diabetes mellitus, thyroid disease), and respiratory (obstructive sleep apnea, asthma, chronic obstructive pulmonary disease [COPD], and other lung diseases)—and a history of SARS-CoV-2 infection was recorded. Comorbid conditions (e.g., hypertension, coronary artery disease, diabetes, COPD, atrial fibrillation) were abstracted from clinician-verified discharge diagnoses and concomitant medication lists at each admission. Because standardized comorbidity severity scales (e.g., Charlson, Elixhauser) were not applied, while comorbidities were modeled as binary indicators (present/absent). Where feasible, key comorbidities (hypertension, coronary artery disease, atrial fibrillation, type 2 diabetes mellitus, and COPD) were included in multivariable models subject to events-per-variable constraints; otherwise, they were summarized descriptively and incorporated into sensitivity analyses.

### 4.7. Follow-Up and Mortality Assessment

Patients were scheduled for evaluations at least every three months, including clinical examination, functional testing, biomarker analysis, and imaging, with additional visits performed in cases of clinical deterioration.

Patients who were lost to follow-up—defined as those who transferred care to another institution, missed scheduled visits, or could no longer be contacted—were excluded from the analysis due to insufficient longitudinal data. Accordingly, only patients with follow-up available per protocol and a complete minimum dataset at scheduled visits were included.

Mortality outcomes included both in-hospital and post-discharge mortality. In-hospital mortality was defined as any death occurring during the index hospitalization. Post-discharge mortality at three months was determined through a review of electronic medical records and structured telephone interviews with patients’ families or caregivers, conducted in compliance with the General Data Protection Regulation (GDPR).

### 4.8. Statistical Analysis

All statistical analyses were performed using R Studio statistical software (Therneau TM. A Package for Survival Analysis in R. R package version 3.8-3, available at: https://CRAN.R-project.org/package=survival (accessed on 17 May 2025)). Continuous variables were tested for normality using the Kolmogorov–Smirnov test. Parametric data are presented as the mean ± standard deviation (SD), while non-parametric continuous variables are reported as median (interquartile range, IQR). Categorical variables are expressed as counts and percentages.

For comparisons between independent groups, the following tests were applied:

- Student’s *t*-test for normally distributed continuous variables,
- Mann–Whitney U test for non-normally distributed continuous variables.

Associations between continuous variables were assessed using:

- Pearson’s correlation coefficient for normally distributed variables,
- Spearman’s rank correlation coefficient for non-parametric variables.

Kaplan–Meier survival analysis with the log-rank test was used to compare survival distributions.

The predictive performance of inflammation-based hematologic indices was assessed using ROC curve analysis with calculation of the AUC. Optimal cut-off values for NLR, PLR, NPAR, LMR, and SII were determined using the maximum Youden index, serving as a basis for future research. In both univariable and multivariable analyses, absolute values were used for all inflammation-based hematologic indices. A *p*-value < 0.05 was considered statistically significant.

Sample size requirements were estimated according to the events-per-variable (EPV) rule proposed by Peduzzi et al., which recommends a minimum of 10 outcome EPV in logistic regression models [44]. Based on the observed in-hospital mortality rates of 12.9% in the PAH subgroup and 15.4% in the CTEPH subgroup, at least 77 PAH and 65 CTEPH admissions were required to ensure stable model estimation with a single predictor. As in-hospital mortality represented the outcome with the fewest events, this endpoint was considered the most conservative for sample size estimation. Consequently, analyses of other endpoints (post-discharge mortality and length of stay) were deemed adequately powered within this framework. Given the very low number of endpoint events in the CTEPH cohort (in-hospital mortality: 4/187; post-discharge mortality: 3/187), Firth’s bias-reduced logistic regression was applied as a sensitivity analysis using a parsimonious set of a priori covariates (age, sex, PH-specific therapy, and comorbidities). Despite multiple simplification and stabilization steps, the Firth models did not yield finite estimates for biomarker terms. Consequently, the results are presented on an exploratory basis, emphasizing effect sizes and 95% CIs from descriptive or minimally adjusted analyses. No claims of independent prognostic value are made for CTEPH.

LOS was treated as a continuous outcome. Distributional assumptions were examined using standard normality tests and Quantile–Quantile (Q–Q) plots, and variance homogeneity was assessed visually and with formal tests. Given the right-skewed and heteroscedastic distribution of LOS, the primary analysis employed a Gamma generalized linear model with a log link. Associations with LOS were also summarized using Spearman’s rank correlation. Sensitivity analyses included linear models fitted to log-transformed LOS and OLS-squares regression with robust standard errors.

To minimize bias and account for inter-cohort heterogeneity, statistical models were constructed separately for PAH and CTEPH, with adjustments for potential confounders including demographic characteristics (age, sex), exposure to disease-specific therapies (endothelin receptor antagonists, phosphodiesterase type 5 inhibitors, soluble guanylate cyclase stimulators), and major comorbidities (hypertension, coronary artery disease, atrial fibrillation, type 2 diabetes mellitus, and chronic obstructive pulmonary disease). Associations between inflammatory indices and clinical outcomes were evaluated within these adjusted models. Confounders were tested against inflammatory indices under different outcome scenarios: LOS (continuous variable), in-hospital mortality, and post-discharge mortality (binary variables). LOS was modeled primarily using a Gamma generalized linear model with a log link; sensitivity analyses included linear models on log-transformed LOS and OLS with HC3 robust SEs.

## 5. Conclusions

This hypothesis-generating study demonstrates that routinely available hematologic inflammation-based indices—particularly NPAR, SII, and NLR—may provide prognostic information in patients with pulmonary arterial hypertension (PAH) and chronic thromboembolic pulmonary hypertension (CTEPH). In PAH, NPAR and SII were associated with in-hospital mortality after adjustment, and NPAR further predicted post-discharge mortality and overall survival. In CTEPH, exploratory associations of NLR, PLR, and SII with in-hospital mortality were observed; however, the very low number of events precluded stable bias-reduced estimation, and these results should therefore be interpreted with caution.

From a pathophysiological perspective, these indices reflect the interplay between the neutrophil–platelet–lymphocyte axis, endothelial injury, and immunothrombotic activation—processes that contribute to pulmonary vascular remodeling, right ventricular dysfunction, and adverse outcomes in both PAH and CTEPH. Clinically, such low-cost, routinely obtainable markers may complement guideline-based risk stratification tools, including REVEAL score and the ESC/ERS 4-strata risk assessment model, by helping to identify patients with heightened inflammatory activity who may benefit from closer monitoring, earlier re-evaluation, or more intensive follow-up. These indices should be regarded solely as adjuncts to established prognostic assessment and not as stand-alone predictors. Prospective, multicenter studies with serial biomarker measurements, predefined cut-offs, and the integration of inflammatory indices with clinical, hemodynamic, and imaging parameters are warranted to validate these findings and explore their potential contribution to personalized risk assessment and management in pulmonary hypertension.

## Figures and Tables

**Table 1:** Baseline characteristics of the PAH and CTEPH cohorts.

Parameter	PAH (*n* = 54)	CTEPH (*n* = 26)	*p*-Value
Age (years, median, IQR)	47.00 (28.50–63.00)	62.50 (56.25–69.00)	<0.001
Sex (female, *n*, %)	35 (64.81%)	11 (42.30%)	0.056
BMI (kg/m^2^, median, IQR)	23.71 (21.22–29.18)	28.15 (25.07–31.52)	0.008
Time from the onset of symptoms to diagnosis (months, median, IQR)	12.00 (6.00–24.00)	12.00 (6.00–13.50)	0.195
Pulmonary Hypertension Group			
IPAH (Group 1.1) (*n*, %)	10 (18.52%)	-	-
CTD-PAH (Group 1.4.1) (*n*, %)	34 (62.96%)	-	-
CHD-PAH (Group 1.4.4) (*n*, %)	10 (18.52%)	-	-
CTEPH (Group 4.1) (*n*, %)	-	26 (100%)	-
WHO-FC (median, IQR)	3.00 (2.00–3.00)	3.00 (2.00–3.00)	0.815
I (*n*, %)	3 (5.56)	1 (3.85)	0.742
II (*n*, %)	20 (37.03)	11 (42.30)	0.650
III (*n*, %)	30 (55.55)	11 (42.30)	0.267
IV (*n*, %)	1 (1.86)	3 (11.55)	0.063
Right heart catheterization data			
mPAP (mmHg, mean ± SD)	50.38 ± 17.65	41.00 ± 10.86	0.030
PAWP (mmHg, median, IQR)	9.50 (6.00–13.00)	11.00 (5.00–15.00)	0.751
PVR (WU, median, IQR)	9.19 (4.01–13.17)	9.00 (6.00–12.00)	0.773
CO (l/min, median, IQR)	4.47 (3.22–5.80)	4.66 (3.52–5.01)	0.609
CI (L/min/m^2^, median, IQR)	2.50 (1.96–3.36)	2.18 (1.80–2.70)	0.069
Four-Strata Risk Assessment (points, median, IQR)	2.00 (1.00–3.00)	2.00 (2.00–3.00)	0.134
Number of Hospital Admissions per patient (count, median, IQR)	4.00 (2.00–7.00)	5.00 (4.00–9.75)	0.058
LOS (days, median, IQR)	6.00 (4.00–8.00)	7.00 (5.00–9.00)	0.002
Overall mortality (*n*, %)	15 (27.78%)	7 (26.92%)	0.936
In hospital mortality (*n*, %)	7 (12.96%)	4 (15.38%)	0.768
Post-discharge mortality (*n*, %)	8 (14.82%)	3 (11.54%)	0.690
Comorbidities			
Cardiovascular Diseases			
Hypertension (*n*, %)	12 (22.22%)	10 (38.46%)	0.128
Coronary heart disease (*n*, %)	3 (5.55%)	4 (15.38%)	0.206
Atrial fibrillation (*n*, %)	13 (24.07%)	8 (30.76%)	0.524
Deep vein thrombosis (*n*, %)	0 (0.00%)	25 (96.15%)	<0.001
Metabolic Disorders			
T2DM (*n*, %)	11 (20.37%)	9 (34.61%)	0.168
Thyroid disease (*n*, %)	15 (27.77%)	4 (15.38%)	0.222
Respiratory Diseases			
Obstructive sleep apnea (*n*, %)	2 (3.70%)	3 (11.53%)	0.323
Asthma (*n*, %)	6 (11.11%)	2 (7.69%)	>0.990
COPD (*n*, %)	4 (7.40%)	5 (19.23%)	0.142
Lung diseases—other than COPD and asthma (*n*, %)	18 (33.33%)	5 (19.23%)	0.192
History of SARS-CoV-2 infection (*n*, %)	14 (25.92%)	7 (26.92%)	0.924

BMI, body mass index; CHD-PAH, PAH associated with congenital heart disease; CI, cardiac index; CO, cardiac output; COPD, chronic obstructive pulmonary disease; CTD-PAH, PAH related to connective tissue disease; CTEPH, chronic thromboembolic pulmonary hypertension; IPAH, idiopathic PAH; IQR, interquartile range; LOS, length of hospital stay; mPAP, mean pulmonary arterial pressure; *n*, number of patients; PAH, pulmonary arterial hypertension; PAWP, pulmonary artery wedge pressure; PVR, pulmonary vascular resistance; T2DM, type 2 diabetes mellitus; WHO-FC, World Health Organization functional class; WU, Wood units. Cardiac output was calculated using the Fick principle.

**Table 2:** Laboratory parameters and biomarker indices.

Parameter	PAH Admissions (*n* = 281)	CTEPH Admissions (*n* = 187)	*p*-Value
Leukocytes (10^3^/µL, median, IQR)	7.13 (5.96–8.45)	6.76 (5.45–8.31)	0.087
Lymphocytes (10^3^/µL, median, IQR)	1.59 (1.23–2.12)	1.44 (1.03–1.88)	0.001
Neutrophils (10^3^/µL, median, IQR)	4.60 (3.70–5.69)	4.52 (3.43–5.80)	0.421
Neutrophils (%, mean ± SD)	66.05 ± 9.26	67.34 ± 9.16	0.138
Monocytes (10^3^/µL, median, IQR)	0.60 (0.50–0.73)	0.67 (0.55–0.79)	0.013
Platelets (10^3^/µL, median, IQR)	207.00 (167.00–243.00)	218.00 (186.00–274.00)	0.001
Albumin (g/dL, median, IQR)	4.10 (3.69–4.46)	4.14 (3.81–4.37)	0.682
Inflammation-based hematologic indices			
NLR (median, IQR)	3.03 (2.10–4.05)	3.01 (2.231–4.62)	0.140
PLR (median, IQR)	122.98 (90.99–183.72)	156.54 (122.87–209.18)	<0.001
NPAR (median, IQR)	16.43 (14.17–19.19)	16.64 (14.86–19.21)	0.431
LMR (median, IQR)	2.63 (1.93–3.65)	2.25 (1.66–2.91)	<0.001
SII (median, IQR)	606.53 (357.59–890.75)	688.95 (494.60–1002.98)	<0.001

IQR, interquartile range; LMR, lymphocyte-to-monocyte ratio; *n*, number of admissions; NLR, neutrophil-to-lymphocyte ratio; NPAR, neutrophil-percentage-to-albumin ratio; PLR, platelet-to-lymphocyte ratio; SII, systemic immune-inflammation index.

**Table 3:** Correlation coefficients between hematologic indices and LOS.

Parameters	PAH Admissions (*n* = 281)		CTEPH Admissions (*n* = 187)	
	r	*p*-Value	r	*p*-Value
NLR	0.178	0.003	0.253	<0.001
PLR	0.142	0.017	0.093	0.205
NPAR	0.174	0.026	0.294	<0.001
LMR	−0.125	0.036	−0.333	<0.001
SII	0.152	0.011	0.18	0.014

LMR, lymphocyte-to-monocyte ratio; *n*, number of admissions; NLR, neutrophil-to-lymphocyte ratio; NPAR, neutrophil-percentage-to-albumin ratio; PLR, platelet-to-lymphocyte ratio; SII, systemic immune-inflammation index.

**Table 4:** Assessment of in-hospital mortality in the study cohort.

Hematologic Indices (Predictors)	PAH Admissions (*n* = 281)				CTEPH Admissions (*n* = 187)			
	OR	95% CI	*p*-Value	Cut-Off Value	OR	95% CI	*p*-Value	Cut-Off Value
NLR	1.582	1.221–2.049	<0.001	>3.28	1.164	0.918–1.477	0.211	>2.96
PLR	1.004	1.000–1.007	0.074	>167.57	1.004	0.995–1.012	0.372	>134.81
NPAR	1.129	1.011–1.261	0.031	>18.98	1.012	0.979–1.045	0.479	>16.13
LMR	0.291	0.108–0.790	0.015	≤1.81	0.227	0.041–1.254	0.089	≤1.30
SII	1.001	1.000–1.002	0.002	>747.32	1.001	1.000–1.002	0.090	>1171.59

LMR, lymphocyte-to-monocyte ratio; *n*, number of admissions; NLR, neutrophil-to-lymphocyte ratio; NPAR, neutrophil-percentage-to-albumin ratio; OR, odds ratio; PLR, platelet-to-lymphocyte ratio; SII, systemic immune-inflammation index.

**Table 5:** Prognostic value of inflammation-based hematologic indices for post-discharge mortality in PAH and CTEPH.

Hematologic Indices (Predictors)	PAH Admissions (*n* = 281)				CTEPH Admissions (*n* = 187)			
	OR	95% CI	*p*-Value	Cut-Off Value	OR	95% CI	*p*-Value	Cut-Off Value
NLR	1.202	0.923–1.566	0.173	>3.56	1.289	1.029–1.615	0.027	>4.83
PLR	1.002	0.998–1.007	0.313	>157.25	1.007	0.998–1.015	0.136	>208.7
NPAR	1.181	1.062–1.313	0.002	>16.86	1.011	0.974–1.050	0.555	>20.18
LMR	0.291	0.108–0.790	0.015	≤2.40	0.227	0.041–1.254	0.089	≤1.98
SII	1.000	0.999–1.001	0.550	>547.5	1.001	1.000–1.002	0.284	>777.55

LMR, lymphocyte-to-monocyte ratio; *n*, number of admissions; NLR, neutrophil-to-lymphocyte ratio; NPAR, neutrophil-percentage-to-albumin ratio; OR, odds ratio; PLR, platelet-to-lymphocyte ratio; SII, systemic immune-inflammation index.

## Data Availability

The data supporting the findings of this study are available from the corresponding author upon reasonable request, in accordance with applicable local and national regulations.

## Abbreviations

The following abbreviations are used in this manuscript:

AUC	Area Under the Curve
BMI	Body Mass Index
CHD	Congenital Heart Disease
CTD	Connective Tissue Disease
CI	Confidence Interval / Cardiac Index
CO	Cardiac Output
COPD	Chronic Obstructive Pulmonary Disease
CHD-PAH	PAH associated with congenital heart disease
CTD-PAH	Connective Tissue Disease-Pulmonary Arterial Hypertension
CTEPH	Chronic Thromboembolic Pulmonary Hypertension
ECG	Electrocardiogram
EF	Ejection Fraction
eGFR	Estimated Glomerular Filtration Rate
EPV	Events-Per-Variable
ESC/ERS	European Society of Cardiology / European Respiratory Society
ESR	Erythrocyte Sedimentation Rate
FC	Functional Class
GDPR	General Data Protection Regulation
GEE	Generalized Estimating Equations
HC3	Heteroskedasticity-Consistent Variant 3
HF	Heart Failure
HR	Hazard Ratio
IPAH	Idiopathic PAH
IQR	Interquartile Range
LMR	Lymphocyte-to-Monocyte Ratio
LOS	Length of Stay
mPAP	Mean Pulmonary Arterial Pressure
NLR	Neutrophil-to-Lymphocyte Ratio
NPAR	Neutrophil-Percentage-to-Albumin Ratio
OLS	Ordinary Least Squares
OR	Odds Ratio
OSA	Obstructive Sleep Apnea
PAH	Pulmonary Arterial Hypertension
PAP	Pulmonary Arterial Pressure
PAWP	Pulmonary Arterial Wedge Pressure
PH	Pulmonary Hypertension
PLR	Platelet-to-Lymphocyte Ratio
PVR	Pulmonary Vascular Resistance
REVEAL	Registry to Evaluate Early and Long-term Pulmonary Arterial Hypertension Disease Management
ROC	Receiver Operating Characteristic
SARS-CoV-2	Severe Acute Respiratory Syndrome Coronavirus 2
SD	Standard Deviation
SII	Systemic Immune-Inflammation Index
T2DM	Type 2 Diabetes Mellitus
WHO	World Health Organization
WHO-FC	World Health Organization Functional Class
WU	Wood Units
WBC	White Blood Cell
